# Correlation between periodontal status, whole salivary interleukin-1beta levels and oral yeasts carriage among individuals with varying ranges of body mass index

**DOI:** 10.2340/aos.v84.43276

**Published:** 2025-03-12

**Authors:** Dena Ali, Toshinari Mikami, Fatema Alkazemi

**Affiliations:** a Department of General Dental Practice, Faculty of Dentistry, Kuwait University, Safat, Kuwait; b Pax Creation Medical Lab, Morioka, Japan; c Department of Oral Pathology, Oral Lab Central College of Stomatology, China Medical University, Shenyang, China

**Keywords:** Alveolar bone loss, body mass index, oral yeasts, whole saliva, periodontal

## Abstract

**Objective:**

The aim was to assess the correlation between periodontal status, whole salivary interleukin-1 beta (IL-1β) levels and oral yeasts carriage (OYC) among individuals with varying ranges of body mass index (BMI).

**Material and method:**

The weight, waist circumference (WC), and height of individuals were assessed. Participants were categorized into three groups: Group-1 – normal weight (18.5–24.9 Kg/m^2^); Group-2 – overweight (25–29.9 Kg/m^2^); and Group-3 – obese (≥ 30 Kg/m^2^). Plaque and gingival indices (PI and GI, respectively), probing depth (PD), clinical-attachment-loss (CAL), missing teeth (MT) and marginal-bone-loss (MBL) were assessed. Whole salivary IL-1β levels and OYC were assessed. Group-comparisons were done. *P* < 0.05 was considered statistically significant.

**Findings:**

Twenty-two, 22 and 22 individuals with comparable mean ages were included in groups 1, 2 and 3, respectively. The mean WC and BMI were higher in Group 3 than Groups 1 (*P* < 0.05) and 2 (*P* < 0.05). The mean PI, CAL, PD, GI, MT and MBL were higher in Group-3 than groups 1 (*P* < 0.05) and 2 (*P* < 0.05). There was no difference in mean PI, CAL, PD, GI, MT and MBL in groups 1 and 2. The salivary flow rate was higher in groups 1 (*P* < 0.05) and 2 (*P* < 0.05) than Group-3. The OYC and IL-1β were higher in Group-3 than groups 1 (*P* < 0.05) and 2 (*P* < 0.05). There was a correlation between PD and OYC in Group-3 (*P* < 0.05).

**Conclusion:**

Periodontal inflammation is worse, and whole salivary IL-1β levels are elevated in obese than overweight individuals and subjects with normal BMI. There seems to be no association between BMI and OYC.

## Introduction

Periodontal disease is a prevalent chronic inflammatory condition affecting the supporting structures of the teeth, namely the gingiva, periodontal ligament, and alveolar bone. The disease progression involves a complex interplay between microbial colonization and host immune response, leading to tissue destruction and eventual tooth loss if left untreated [1, 2]. The primary risk factor associated with periodontal inflammatory conditions, including periodontitis, is commonly acknowledged to be poor oral hygiene practices [3]. However, extensive scientific evidence from indexed databases indicates that there is a bidirectional association between systemic and periodontal health statuses of susceptible individuals [4–7].

An increased body mass index (BMI), particularly in overweight (25-29.9 Kg/m2) and obese (≥ 30 Kg/m2) individuals, is a recognized risk factor for periodontal diseases [8–10]. Results from an observational study [11] conducted in Riyadh, Saudi Arabia showed significantly higher scores of plaque index (PI) and gingival index (GI) among overweight and obese individuals compared with normal-weight individuals. Likewise, in another study from Egypt, a statistically significant correlation was reported between BMI and waist circumference (WC) and scores of GI and clinical attachment loss (CAL) [9]. The precise mechanism through which an increased BMI affects periodontal tissues remains a subject of debate; however, it has been reported that adipose tissue in individuals with an increased BMI is characterized by chronic low-grade inflammation, with increased expression of pro-inflammatory cytokines such as interleukin-1 beta (IL-1β) and tumor necrosis factor-alpha (TNF-α) in the serum and unstimulated whole saliva (UWS) [12, 13]. These cytokines are known to contribute to local and systemic inflammation, including within the periodontium [14]. Moreover, an elevated BMI has also been linked to impaired healing processes, which may further compromise periodontal tissue repair and regeneration in vulnerable individuals [15]. Under physiological conditions, oral yeasts (predominantly *Candida* species) reside in the oral cavity maintaining a symbiotic relationship with other microbes; however, local and systemic factors such as tobacco smoking and a state of persistent hyperglycemia enhance oral yeasts carriage (OYC) thereby increasing the vulnerability of individuals to oral diseases such as candidiasis [16, 17]. It has also been reported that an increased OYC is more often manifested in patients with than those without periodontitis [18]. Although it has been suggested that there is a potential association between an increased BMI and OYC, the available evidence is not yet definitive [19]. However, according to Nawaz et al. [20], an increased expression of pro-inflammatory cytokines in UWS such as IL-1β is associated with an increased OYC. Therefore, there is a likelihood that an augmented whole salivary immunoinflammatory response among patients with elevated BMI jeopardizes periodontal tissues and increases OYC as well as IL-1β levels in these individuals compared with individuals with a normal BMI. The present study is based on the hypothesis that periodontal inflammation is worse and whole salivary OYC and IL-1β levels are elevated among patients with higher BMI in contrast to individuals with BMI within the normal range.

## Aim

The aim was to assess the correlation between periodontal status, whole salivary IL-1β levels and OYC among individuals with varying ranges of BMI.

## Methods

### Ethical approval

The research study received approval from the Ethics Committee of the Health Science Center (HSC), Kuwait University (VDR/EC-734). Participation was voluntary, and individuals could decline or withdraw at any time. Those who chose to volunteer were required to sign a consent form. Prior to participation, all individuals were provided with detailed information regarding the study’s objectives and methodology, and they were encouraged to ask any questions they had.

### Study design, dates and location

The present study has an observational cohort design and was performed at the Comprehensive Dental Clinic, Faculty of Dentistry, Kuwait University, Kuwait between June 2024 and December 2024.

### Eligibility criteria

The inclusion criteria were as follows: (a) adult males and females at least 18 years old; (b) normal weight individuals (BMI 18.5 to 24.9 Kg/m^2^); (c) overweight individuals (25.0 to 29.9 Kg/m^2^); (d) obese individuals (≥ 30.0 Kg/m^2^). From a dental standpoint, individuals who were completely edentulous, those with impacted third molars, severely decayed or fractured teeth containing embedded root fragments, and those with supernumerary teeth were excluded from the study. In terms of systemic health considerations, individuals who self-reported diabetes mellitus (DM), cardiovascular ailments, respiratory conditions, hepatic and/or renal disorders, as well as those diagnosed with HIV/acquired immune deficiency syndrome, were not included in the sample sought for analysis.

### Study groups

The BMI was determined by dividing weight (in kilograms) by the square of height (in meters) and expressed as kilograms per square meters (Kg/m^2^). According to the criteria from the Centers for Disease Control and Prevention, participants were categorized into three groups as follows: Group-1 – normal weight (18.5–24.9 Kg/m^2^); Group-2 – overweight (25–29.9 Kg/m^2^); and Group-3 – obese (≥ 30 Kg/m^2^) [21].

### Demographic data and education status

The questionnaire was designed to be clear and easy to understand to facilitate accurate responses. One investigator conducted the data collection to maintain consistency and minimize bias. A structured questionnaire was developed, including sections for demographic information and oral hygiene practices. Demographic variables, namely age, gender, and education status were self-reported by participants. Oral hygiene practices, including daily toothbrushing frequency (once or twice daily), flossing habits (once or twice daily), and the last dental visit (within the past 6 months, between 6 and 12 months, and over a year ago) were also self-reported. Individuals who indicated completion of education up to the 10th grade were categorized as having achieved ‘School-level education’ [22], and participants who had completed an additional at least 24-months of college education after finishing their school education were categorized as having ‘College-level education’ [22]. Individuals who reported graduation from a university after completing college-level education were classified as having ‘University-level education’ [22].

### Assessment of body mass index and waist circumference

A standardized protocol, as described elsewhere, was adopted for conducting anthropometric measurements [23]. A trained and calibrated investigator (kappa score 0.88) assessed the height (in meters), weight (in kilograms), and WC (in centimeters) of all participants. Measurements for height, weight, and WC were taken using specific equipment: a portable digital weighing scale, stadiometer (Prodoc Detecto PD300, Cardinal Scale Manufacturing Co., MO USA), and a constant tension tape. These parameters were measured with the patients wearing light attire and shoes off. WC was assessed at the midpoint between the lower rib cage and the iliac crest following a full exhalation. The BMI was computed as weight divided by the square of height and expressed as Kg/m^2^.

### Clinical and radiographic evaluations

A full mouth clinical periodontal assessment was done before the collection of UWS samples and assessment of OYC. Clinical (Kappa score 0.9) and radiographic (Kappa score 0.86) evaluations were carried out by a calibrated investigator. Full-mouth PI [24], probing depth (PD) [25] and GI [26] were evaluated at the buccal, lingual/palatal, mesial, and distal surfaces on all teeth. The CAL [27] and PD were measured to the nearest millimeter using a graded probe (Hu-Friedy InC, Chicago, IL, USA). Full-mouth digital intra-oral radiographs were taken (Planmeca Romexis Intra oral X-Ray, Planmeca OY, Helsinki, Finland), with standardization procedures applied as previously described. The number of missing teeth (MT) was also documented.

### Collection of whole saliva

The UWS samples were collected using a protocol described in a previous investigation [28]. In summary, fasting whole saliva samples were collected by a calibrated investigator (Kappa score 0.9) with patients seated comfortably on a chair in a quiet room. Patients were instructed to allow saliva to accumulate in their mouths for 5 min and refraining from swallowing or moving their jaw during this period. The collected UWS was then expectorated into a measuring cylinder, and the salivary flow rate was determined. Subsequently, each saliva sample was transferred to sterile plastic tubes and securely sealed (Salivette™, Sarstedt Inc., Numbrecht, Germany). The supernatant was extracted by vortexing the tubes at 1500 × g for a continuous 5-min period. Following extraction, the supernatant was stored at −70°C and evaluated within 24 h.

### Assessment of whole salivary interleukin 1 beta levels

The whole salivary IL-1β levels were assessed as described by Majeed et al. [29] In summary, IL-1β levels were determined using the enzyme-linked immunosorbent assay via commercially available kits (SolarBio Science & Technology Co, Ltd., Beijing, China), which were used according to instructions from the manufacturer. Each sample contributed 10 μL of saliva into designated wells of a 96-well plate in duplicates, pre-filled with 40 μL of sample diluent provided with the kit. Following this, the plates were incubated at 37°C for 30 min, and then 10 μL each of Chromatin Solution A and Chromatin Solution B were added. The process was finalized by adding 50 μL of stop solution to each well. After incubating in the dark for 15 min, the microplates were examined using a reader at 450 nm (ELX808, BioTek, Santa Clara, CA, USA). The assay’s sensitivity was confirmed to be less than 0.1 pg/mL, ensuring detection of cytokines at low concentrations, and no cross-reactivity was observed with a wide range of related cytokines. All samples were assessed by a trained and calibrated investigator (Kappa score 0.9) blinded to the study groups.

### Evaluation of oral yeasts carriage

The OYC was assessed an hour after the collection of UWS samples using the oral rinse technique [30]. Participants were provided with 10 mL of sterile phosphate-buffered saline (PBS 0.01 M, pH 7.2) in a sterile disposable universal container and instructed to rinse for 1 min. Subsequently, the rinse was expectorated back into the same container. Samples were promptly transported to the microbiology laboratory for mycological analysis. Upon arrival, oral rinse samples underwent centrifugation at 3000 rpm (Jouan C412, France) for 10 min. The supernatant was discarded, and one mL of sterile PBS was added to the sediment, which was then agitated on a bench vibrator (VibroFix VF1 electronic, Italy) for 30 s to disaggregate microorganisms. Following this, 0.5 mL of the processed mixture was inoculated onto Sabouraud’s dextrose agar plates (Oxoid Ltd, Basingstoke, Hampshire, England). The plates were aerobically incubated at 37°C for 48 h, followed by an additional 24 h at room temperature to optimize fungal growth. Colony counting was performed on the plates of oral rinse cultures, and the resulting count was multiplied by two to determine the number of colony-forming units per ml of the rinse (CFU/ml) [30].

### Sample-size estimation and statistical analysis

Sample-size estimation (SSE) was done using data from a pilot investigation. The preliminary SSE was founded on the variation in PD alteration. Based on a recent cross-sectional study [31], a disparity of 0.5 mm was deemed to be clinically significant. Following a power analysis involving 66 participants (22 per group) and a significance level (α) of 0.05, it was determined that there was a 90% probability of identifying a genuine difference of 0.5 mm. There were no foreseen drop-outs. A software program (IBM, SPSS, Version 22, Chicago, IL, USA) was used to perform quantitative evaluations. Group-comparisons were done using one-way analysis of variance and Bonferroni post-hoc adjustment tests. Regression analysis was also carried out to determine the correlation between BMI and clinicoradiographic, whole salivary IL-1β and OYC. P<0.05 were considered statistically significant.

## Findings

### Recruitment of study participants

**Figure 1:**
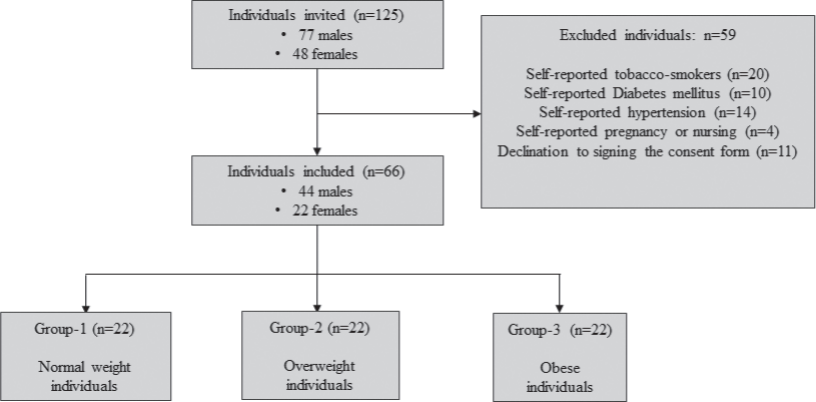
Recruitment of participants.

One hundred and twenty-five individuals (77 males and 48 females) were invited to participate in the present study. Amongst the male volunteers, 20 self-reported tobacco-smokers, 10 individuals with self-reported DM and three individuals on medications for hypertension were excluded. Twenty-six females were excluded for the following reasons: (a) pregnant/nursing (*n* = 4), (b) self-reported hypertension (*n* = 11) and (c) declining to sign the written consent form (*n* = 11). In total, 66 individuals (44 males and 22 females) were included in the present investigation (Figure 1).

### Demographics of the study cohort

Twenty-two, 22 and 22 individuals were included in groups 1, 2 and 3, respectively. In groups 1, 2 and 3, 17, 15 and 12 individuals, respectively were males. The mean ages of individuals in groups 1, 2 and 3 were 43.1 ± 7.6, 46.8 ± 9.1 and 50.3 ± 6.7 years, respectively. The mean WC was significantly higher among individuals in groups 2 (*P* < 0.05) and 3 (*P* < 0.05) compared with individuals in Group-1 (*P* < 0.05). The mean WC was significantly higher among individuals in Group-3 compared with individuals in Group-2 (*P* < 0.05). The mean BMI was significantly higher among individuals in groups 2 (*P* < 0.05) and 3 (*P* < 0.05) compared with individuals in Group-1 (*P* < 0.05). The mean BMI was significantly higher among individuals in Group-3 compared with individuals in Group-2 (*P* < 0.05). University-level education was attained by 68.2%, 54.5% and 18.2% individuals in groups 1, 2 and 3, respectively. Toothbrushing twice daily was more often reported by individuals in groups 1 (81.8%) and 2 (100%) than those in Group-3 (13.6%). Ten (45.5%) and 18 (81.8%) individuals in groups 1 and 2, respectively reported that they performed flossing of interproximal spaces once daily. None of the individuals in Group-3 reported to have ever used dental floss. Thirteen (59.1%) and 17 (77.3%) individuals in groups 1 and 2, respectively reported that they had visited a dentist/dental hygienist within the past six to 12 months. All individuals in Group-3 reported that they had last visited a dentist/dental hygienist over a year ago (Table 1).

**Table 1:** General characteristics of study cohort.

Parameters	Group-1	Group-2	Group-3
Participants (*n*)	22	22	22
Gender (male : female)	17 : 5	15 : 7	12 : 10
Mean age (in years)	43.1 ± 7.6 years	46.8 ± 9.1 years	50.3 ± 6.7 years
Mean weight (Kg)	74.5 ± 6.5 Kg^*†^	94.6 ± 3.4 Kg^†^	125.8 ± 3.1 Kg
Mean height (m)	1.79 ± 0.08 m	1.78 ± 0.06 m	1.72 ± 0.08 m
Waist circumference (cm)	83.1 ± 3.5 cm^*†^	98.5 ± 2.1 cm^†^	130.1 ± 6.5 cm
Mean BMI (Kg/m^2^)	23.3 ± 0.8 Kg/m^2*†^	30.1 ± 3.2 Kg/m^2†^	43 ± 1.8 Kg/m^2^
School-level education	None	None	None
College-level education	7 (31.8%)	10 (45.5%)	18 (81.8%)
University level education	15 (68.2%)	12 (54.5%)	4 (18.2%)
Toothbrushing (once daily)	4 (18.2%)	None	19 (86.4%)
Toothbrushing (twice daily)	18 (81.8%)	22 (100%)	3 (13.6%)
Flossing (once daily)	10 (45.5%)	18 (81.8%)	None
Flossing (twice daily)	0	0	None
No flossing	12 (54.5%)	4 (18.2%)	22 (100%)
Visit to dentist/hygienist			
Within 6-months	5 (22.7%)	5 (22.7%)	0
Within 6 to 12 months	13 (59.1%)	17 (77.3%)	0
Over a year ago	4 (18.2%)	0	22 (100%)

BMI: body mass index; cm: centimeters; m: meters; Kg: kilograms; Kg/m^2^: kilograms per square meters.

* Compared with Group-2 (*P* < 0.05).

† Compared with Group-3 (*P* < 0.05).

### Periodontal status and missing teeth

The mean PI, CAL, PD, GI, MT and marginal-bone-loss (MBL) (mesial and distal) were significantly higher in Group-3 compared with groups 1 (*P* < 0.05) and 2 (*P* < 0.05). There was no difference in the mean PI, CAL, PD, GI, MT and MBL (mesial and distal) among individuals in groups 1 and 2 (Table 2).

**Table 2:** Clinical and radiographic periodontal status.

Parameters	Group-1	Group-2	Group-3
Participants (*n*)	22	22	22
Plaque index	0.35 ± 0.2*	0.45 ± 0.3*	0.81 ± 0.1
Gingival index	0.32 ± 0.3*	0.38 ± 0.3*	0.83 ± 0.2
Probing depth (mm)	1.37 ± 0.5 mm*	1.75 ± 0.7 mm*	4.7 ± 0.8 mm
Clinical attachment loss	0.22 ± 0.2 mm*	0.4 ± 0.3 mm*	3.7 ± 0.7 mm
Marginal bone loss (mesial)	1.3 ± 0.6 mm*	1.4 ± 0.6 mm*	4.2 ± 0.7 mm
Marginal bone loss (distal)	1.1 ± 0.7 mm*	1.3 ± 0.7 mm*	4.3 ± 0.8 mm
Missing teeth	1.5 ± 1.4 teeth*	1.72 ± 1 teeth*	6.5 ± 1.8 teeth

* Compared with Group-3 (*P* < 0.05).

### Salivary flow rate, interleukin 1-beta levels and oral yeasts carriage

The SFR was significantly higher in groups 1 (0.3 ± 0.1 ml/min) (*P* < 0.05) and 2 (0.3 ± 0.08 ml/min) (*P* < 0.05) compared with Group-3 (0.07 ± 0.01 ml/min). There was no statistically significant difference in the SFR among individuals in groups 1 (0.3 ± 0.1 ml/min), and 2 (0.3 ± 0.08 ml/min). Whole salivary IL-1β levels were significantly higher in Group-3 (233.1 ± 79.2 pg/ml) compared with groups 1 (43.2 ± 18.2 pg/ml) (*P* < 0.05) and 2 (52.2 ± 18 pg/ml) (*P* < 0.05). There was no difference in whole salivary IL-1β levels among individuals in groups 1 (43.2 ± 18.2 pg/ml) and 2 (52.2 ± 18 pg/ml). The OYC was significantly higher in Group-3 (910.3 ± 216.6 CFU/ml) compared with groups 1 (304.3 ± 104.2 CFU/ml) (*P* < 0.05) and 2 (384.3 ± 49.2 CFU/ml) (*P* < 0.05). There was no difference in OYC among individuals in groups 1 (304.3 ± 104.2 CFU/ml) and 2 (384.3 ± 49.2 CFU/ml).

### Correlation between body mass index and periodontal parameters, salivary interleukin 1-beta and oral yeasts carriage

#### Body mass index. Probing depth, Salivary interleukin 1-beta and oral yeasts carriage

**Figure 2:**
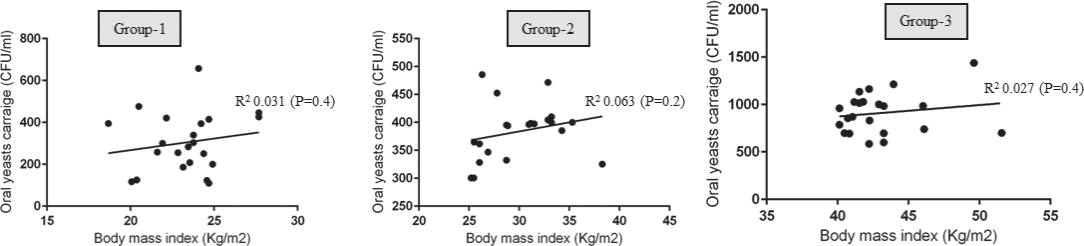
Correlation between body mass index and oral yeasts carriage in groups 1, 2 and 3.

**Figure 3:**
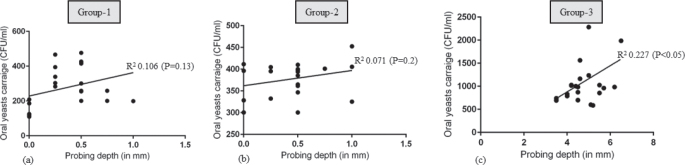
Correlation between probing depth and oral yeasts carriage in groups 1, 2 and 3.

**Figure 4:**
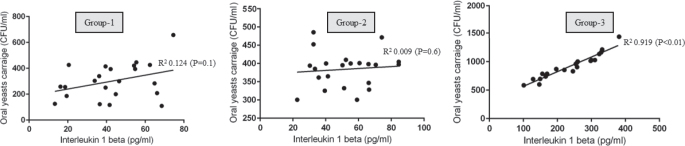
Correlation between whole salivary interleukin 1-beta and oral yeasts carriage in groups 1, 2 and 3.

**Figure 5:**
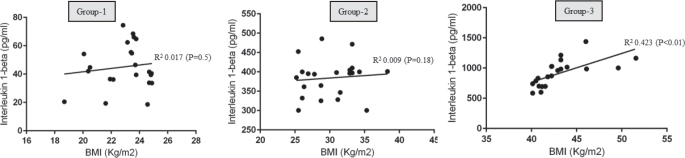
Correlation between body mass index and whole salivary interleukin 1-beta in groups 1, 2 and 3.

**Figure 6:**
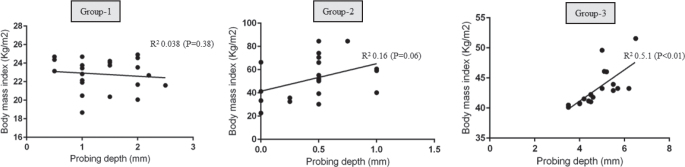
Correlation between probing depth and body mass index in groups 1, 2 and 3.

There was no statistically significant correlation between BMI and OYC in groups 1, 2 and 3 (Figure 2). There was a statistically significant correlation between PD and OYC among individuals in Group-3 (*P* < 0.05) (Figure 3). There was no correlation between PD and OYC among individuals in groups 1 and 2 (Figure 3). There was a statistically significant correlation between whole salivary IL-1β levels and OYC among individuals in Group-3 (Figure 4). There was no correlation between whole salivary IL-1β levels and OYC among individuals in groups 1 and 2 (Figure 4). There was a statistically significant correlation between IL-1β and BMI among individuals in Group-3. There was no statistically significant correlation between IL-1β and BMI among individuals in groups 1 and 2 (Figure 5). There was a statistically significant correlation between PD and BMI among individuals in Group-3. There was no statistically significant correlation between PD and BMI among individuals in groups 1 and 2 (Figure 6). There was no statistically significant correlation between age, gender, PI, GI, CAL, MBL, MT, WC, and education status and OYC and IL-1β levels (data not shown).

## Discussion

The 2007 U.S. National Health and Nutrition Examination Survey (NHANES) reported that 63% of Americans are presently classified as overweight, while 26% fall into the obese category [32]. The present study was performed in the State of Kuwait (Kuwait), which has a population of approximately 4.3 million residents. It is worth mentioning that Kuwait has one of the highest obesity rates on a global scale with prevalence rates for overweight and obesity being 40.6% (95% confidence interval [CI]: 38.4–42.8%) and 42.1% (95% CI: 40.0–44.3%), respectively in the year 2021 [33]. Such alarming results in turn suggest that Kuwaiti nationals with a BMI surpassing the normal range (18.5 to 24.9 Kg/m^2^) face an increased likelihood of either developing periodontal disease and/or experiencing compromised periodontal health. In a systematic review and meta-analysis of five interventional and eight longitudinal studies, Keller et al. [34] demonstrated that increased BMI and WC are significant risk factors of periodontal disease. In a recent case-control study, Shoukat et al. [35] reported that an elevated BMI is a risk factor for increased yeast carriage in the gut in contrast to controls (individuals with a normal BMI); however, the potential relationship between BMI and OYC remains a subject of debate. In this context, the authors of the present observational cohort study hypothesized that periodontal disease and increased OYC are more often manifested in individuals with a higher BMI (overweight and obese) in contrast to controls.

The present results showed that although OYC was significantly higher among individuals in Group-3 (obese individuals) compared with individuals in groups 1 and 2 (normal weight and overweight individuals, respectively), there was no statistically significant correlation between BMI and OYC in all groups. This suggests that an increased BMI does not independently influence OYC; rather, there are likely other contributing factors within the obese population studied. Among these factors, the most significant appears to be the SFR. It is worth noting that the SFR was significantly higher among patients in groups 1 and 2 in contrast to Group-3. The authors of the present investigation applaud previous studies [36, 37], which showed that hyposalivation is a risk factor for increased OYC. Interestingly, a statistically significant correlation was observed between OYC and IL-1β levels among individuals in Group-3. The IL-1β is chiefly produced by mononuclear phagocytes and plays a critical role in mediating immune responses to inflammatory insults [38]. According to Steele and Fidel [39], an increased OYC also results in an increased production of IL-1β. One reasoning for this is that an increased IL-1β production augments the ‘killing’ efficacy of neutrophils thereby driving a protective immunity [40]. It is also worth mentioning that whole salivary IL-1β levels were markedly lower in groups 1 and 2 than Group-3, and the current mycological results demonstrated that OYC was also lower in the former groups than Group-3. These findings indicate that a reduced SFR creates a favorable oral environment conducive to OYC; and in response, the immune system ramps up the production of IL-1β to mitigate the disturbance in the oral microbial equilibrium. Based on the current findings, the authors hypothesize that a higher BMI alone does not directly influence OYC, suggesting that factors such as SFR may play a more prominent role in this context. Future research should focus on longitudinal studies to further investigate the interplay between BMI, SFR, and OYC. Specifically, controlled studies with larger sample sizes and diverse populations should be conducted to assess whether SFR mediates the relationship between BMI and OYC. Moreover, exploring potential biological mechanisms, such as metabolic and inflammatory pathways, may provide deeper insights into how systemic factors contribute to OYC.

From a clinical and radiographic perspective, the present study results differ from previous studies [34, 41], which showed that periodontal inflammation is worse in overweight and obese individuals than those with BMI within the normal range. Interestingly, the present results showed no difference in clinical and radiographic parameters (PI, GI, CAL, MT, PD and MBL, respectively) among individuals in groups 1 and 2. One explanation for this is that the normal and overweight individuals who volunteered to participate in the present investigation seemed to be more responsible towards oral hygiene status (OHS) as toothbrushing twice daily was reported by at least 80% and 100% of individuals in groups 1 and 2, respectively. Moreover, flossing of interproximal spaces once daily was reported by nearly 50% and 81% of individuals in groups 1 and 2, respectively. Furthermore, individuals in groups 1 and 2 reported that they visited their oral healthcare providers (OHPs) on a routine basis. These factors seem to have contributed towards maintenance of periodontal health status and retention of most of the natural dentition in contrast to obese patients who reported to have never used dental floss, mostly brushed once daily and visited an OHP at least over a year ago. The authors of the present study speculate that routine oral hygiene maintenance coupled with routine dental visits seem to have played a role in reducing OYC and salivary IL-1β levels among individuals in groups 1 and 2; and the contribution of a superior education status in these individuals (in contrast to Group-3 where university-level education was less prevalent) towards maintaining oral health routinely cannot be overlooked. The authors applaud the study by Muzurovic et al. [42] according to which, a compromised OHS is a well-known risk factor of increased OYC and associated diseases such as candidiasis.

### Strengths and limitations

Based on the present study results, it is highly recommended that community-based oral health awareness programs should routinely be conducted to educate the masses about the importance of oral and systemic health maintenance and the linkage between the two. A limitation of the present investigation is that the laboratory-based immune-inflammatory investigations were limited to the assessment of whole salivary IL-1β levels. It is hypothesized that other inflammatory biomarkers such as IL-6 and TNF-α are also elevated and possibly correlated with clinicoradiographic parameters and OYC in overweight and obese individuals in contrast to controls (normal weight individuals). Moreover, habitual nicotine product users and patients with other systemic diseases such as DM were excluded from the present study. The primary reason for this was that habitual smoking and DM are independent, yet significant risk factors of increased OYC and periodontal and peri-implant inflammatory conditions [43–45]. Therefore, the inclusion of participants with such characteristics could have potentially biased the present results. Further power-adjusted and well-designed studies are needed to substantiate these hypotheses.

## Conclusion

Periodontal inflammation is worse and whole salivary IL-1β levels are elevated in obese compared with overweight individuals and subjects with a normal BMI. An elevated BMI does not seem to influence OYC in vulnerable populations.
